# Excessive weight loss during alternate day modified fasting increases the risk of hyperuricemia and muscle loss

**DOI:** 10.3389/fnut.2026.1842818

**Published:** 2026-09-09

**Authors:** Yanjuan Zhao, Tingting Yang, Wen Wang, Jing Wen, Yan Qiao, Zhi Zou, Jing Zhou, Xiaoling Wu, Yongchun Chen, Yongli Li

**Affiliations:** 1 Department of Clinical Nutrition, Henan Provincial People's Hospital and Zhengzhou University People's Hospital, Zhengzhou, Henan, China; 2 Department of Infection Management, Tianjin Union Medical Center, The First Affiliated Hospital of Nankai University, Tianjin, China; 3 Department of Medical Imaging, Henan Provincial People's Hospital and Zhengzhou University People's Hospital, Zhengzhou, Henan, China; 4 Department of Nephrology, Henan Provincial Clinical Research Center for Kidney Disease, Henan Provincial Key Laboratory of Kidney Disease and Immunology, Henan Provincial People's Hospital and Zhengzhou University People's Hospital, Zhengzhou, Henan, China; 5 Department of Nuclear Medicine, Henan Provincial People's Hospital and Zhengzhou University People's Hospital, Zhengzhou, Henan, China

**Keywords:** alternate day modified fasting, body composition, muscle loss, obesity, uric acid

## Abstract

**Background:**

Obesity has become a significant contributor to non-communicable diseases worldwide, making it imperative to explore effective weight-loss methods. Alternate day modified fasting (ADMF) has emerged as a popular approach, but its effects on uric acid (UA) dynamics and body composition in Chinese populations remain unclear. This study aimed to investigate whether ADMF could change body composition and cardiometabolic parameters in individuals with obesity, and to analyze the correlation between changes in body composition indices and UA levels.

**Methods:**

A total of 35 adults were recruited and completed a 62-day trial, which consisted of a 32-day high-controlled fasting phase (HCFP) and a 30-day low-controlled fasting phase (LCFP). Body composition was measured by bioelectrical impedance analysis, and blood pressure (BP) and metabolic parameters were assessed at baseline, day 16, day 32, and day 62.

**Results:**

Of the 35 participants (20 males, 15 females; mean age 36.23 ± 8.98 years; baseline body weight 100.02 ± 16.89 kg; baseline BMI 35.47 ± 4.15 kg/m^2^), body weight decreased significantly over the entire 62-day ADMF intervention (absolute loss: −6.83 ± 4.26 kg; −7.02%, *P* < 0.001). The proportion of total weight loss attributable to fat free mass (FFM) was 22.68%. During HCFP, weight decreased by −6.38% (absolute loss: −6.29 ± 3.11 kg, *P* < 0.001), with significant reductions in body fat mass (BFM, −11.14%, *P* < 0.001), FFM (−3.32 %, *P* < 0.001), visceral fat area (VFA, −13.10%, *P* < 0.001), and waist circumference (WC, −5.35%, *P* < 0.001). However, significant losses in soft lean mass (SLM; −3.40%, *P* < 0.001) and fat free mass (FFM; −3.32%, *P* < 0.001) were observed during HCFP, with partial improvement during LCFP (SLM: +0.73%, *P* = 0.031; FFM: +0.78%, *P* = 0.022). A transient but significant increase in UA occurred during HCFP (+27.30 ± 79.60 umol/L, +8.85% from baseline, *P* = 0.047), which subsequently decreased during LCFP (−33.70 ± 72.48 μmol/L, −6.67% from Day 32 to Day 62, *P* = 0.012). This UA elevation was correlated with concurrent loss of SLM (*r* = −0.340, *P* = 0.025).

**Conclusion:**

A phased ADMF intervention was associated with effective reduction in adiposity and improvement in cardiometabolic risk factors. However, the transient hyperuricemia and lean muscle loss during the intensive phase suggest that UA monitoring and muscle-preserving strategies may be considered to optimize the safety and efficacy of ADMF protocols.

## Introduction

Obesity has become an increasingly serious public health problem in China and worldwide, due to its increasing the risk of cardiovascular diseases, cancer and other metabolic diseases (1–3). According to the specific criteria for Chinese individuals, 34.3% of Chinese adults were overweight (≥18 years), and 16.4% were obese (4). In 2019, 11.1% of the total deaths caused by non-communicable diseases (NCDs) were due to overweight or obesity; this is a significant increase compared with the rate of 5.7% in 1990 (4). These situations not only result in threats to personal health but also greatly increase the national health expenditure for the management of NCDs (5, 6).

Reducing energy intake through dietary restriction has been proven to reduce the risk of NCDs in participants with obesity (7). In a previous study, the most common form of dietary restriction was daily caloric restriction (CR), which required individuals to reduce their energy intake by 15–40% of the baseline requirement every day (8). Although CR has been proven to be effective at reducing body weight, many populations with obesity believe that its weight loss effect is slow and its restrictions are not easy to adhere to. Another form of dietary restriction that has become a popular method of weight loss is alternate day fasting (ADF) (9). For the population with obesity, ADF could improve compliance with dietary restrictions because these programs need to limit energy intake only every other day, rather than every day, such as during the CR. The ADF program involves alternating between a “feeding day” (24 h of *ad libitum* food intake) and a “fast day” (24 h of complete fasting). An modified ADF (ADMF) scheme has also been implemented, allowing 20–25% of the energy requirement to be consumed on fasting days (10).

Previous studies demonstrated that ADMF could effectively reduce body weight and visceral fat mass in individuals with overweight and obesity. In addition, favorable changes have been observed regarding risk factors for coronary heart disease, such as reductions in total cholesterol (TC) and low-density lipoprotein cholesterol (LDL), decreased blood glucose levels, and increased insulin sensitivity (9, 11–13). Moreover, a previous study by Trepanowski et al. (14) showed that body weight and blood lipid levels were more favorable during the ADMF weight loss phase; nevertheless, during the ADMF weight maintenance phase, LDL was significantly increased. However, research on ADMF among individuals with obesity in China is limited, and there is a dearth of studies examining the variations in uric acid (UA) levels during ADMF-induced weight loss, which has been proved to be associated with the occurrence of gout and cardiovascular disease (15). To address this gap, the present study endeavors to conduct a 62-day ADMF intervention, aiming to observe the impact of ADMF on the body composition and cardiac metabolic parameters of individuals with obesity in China, and to explore the correlation between UA fluctuations and alterations in body composition indices.

## 2 Material and methods

### 2.1 Study participants

**Figure 1:**
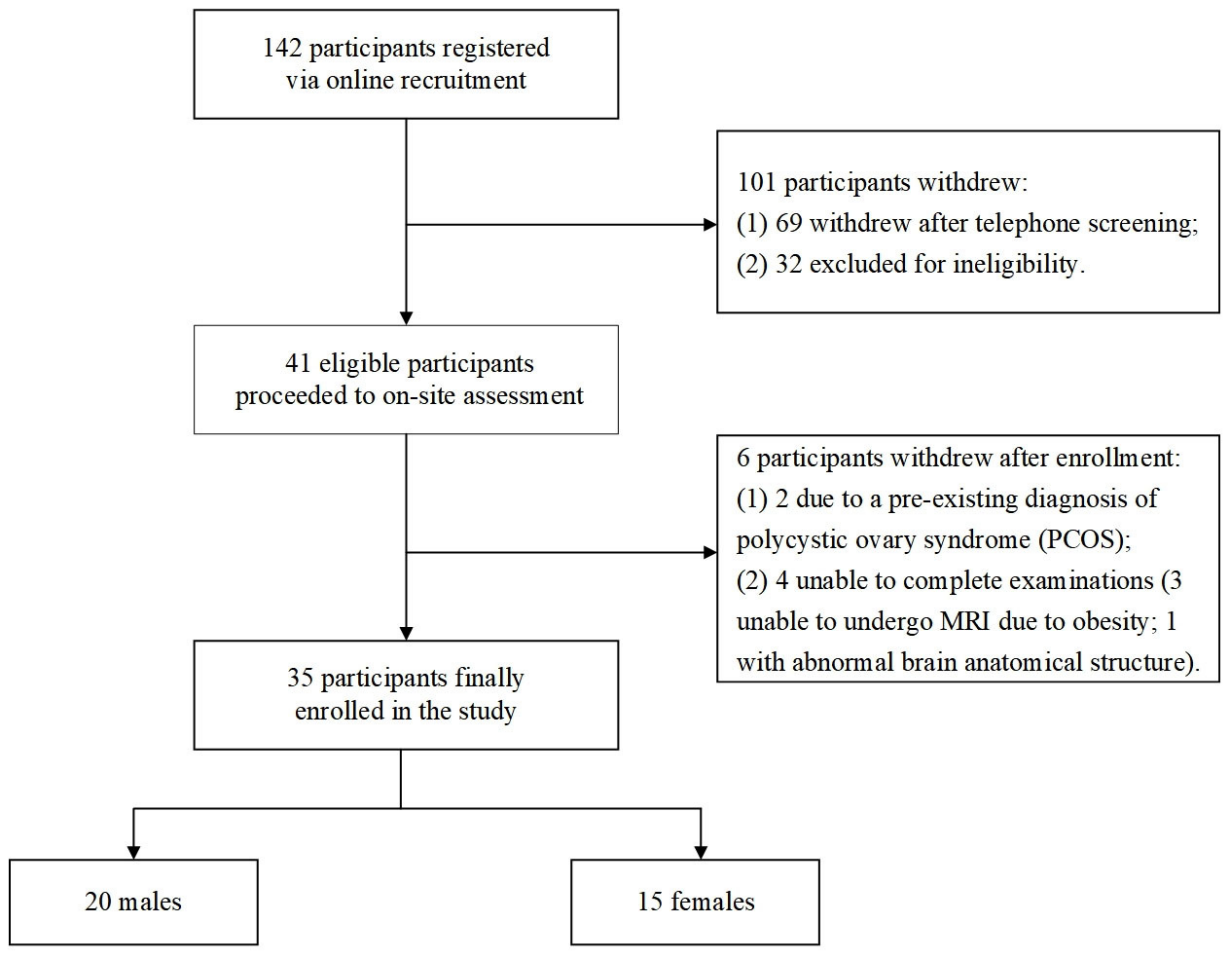
Flow chart of participants through the study.

Volunteers were recruited from a tertiary hospital in Zhengzhou, Henan Province, China. Participants were included if they met the following inclusion criteria: (1) aged 18–60 years; (2) met the definition for obesity (BMI ≥28 kg/m^2^) indicated by the Working Group on Obesity in China (WGOC) (16); and (3) had a waist circumference ≥ 90 cm for males and ≥ 85 cm for females. Participants were excluded if they met any of the following criteria: (1) had a history of serious cardiopathy including New York Heart Association (NYHA) class III-IV heart failure, uncontrolled arrhythmias, or myocardial infarction within the past 6 months. Hypertensive cardiomyopathy without heart failure was not excluded. (2) had chronic liver disease including Child-Pugh class B or C, or active hepatitis. Non-alcoholic fatty liver disease without significant liver dysfunction was not excluded. (3) had chronic kidney disease, defined as estimated glomerular filtration rate (eGFR) < 60 mL/min/1.73 m^2^. (4) had cancer, hematopoietic and systemic immune system disease, infectious disease or nervous system disease. (5) had a history of taking steroids or hormonal agents. (6) were pregnant or planning pregnancy in the next 2 months. (7) had received weight loss therapy such as GLP-1 RA and other drugs in the past half year; (8) could not be measured for body composition using BIA because they contained metal substances that could not be removed. In addition, the participants with pre-existing hypertension, diabetes, hyperlipidemia who were on stable medication (defined as no dose change in the past 3 months) were allowed to participate, provided their conditions were well-controlled. For participants with pre-existing hyperuricemia, only those who were not using urate-lowering therapy were allowed to participate. The participant flow chart was illustrated in Figure 1. A total of 142 individuals initially expressed interest via online recruitment. After telephone screening, 41 eligible participants proceeded to on-site assessment. Of these, 6 withdrew after enrollment: 2 due to a pre-existing diagnosis of polycystic ovary syndrome (PCOS), which may influence metabolic outcomes, and 4 who were unable to complete the required examinations (3 could not undergo MRI scanning due to obesity; 1 had abnormal brain anatomical structure). Ultimately, 35 participants were enrolled and completed the 62-day trial. All the responders provided informed consent and voluntarily participated in the study. The experimental protocol was approved by the ethics committee of Henan Provincial People's Hospital [No. 2018(18)] and registered at clinicaltrials.gov (Protocol ID: 20180520).

### 2.2 Study design

**Figure 2:**
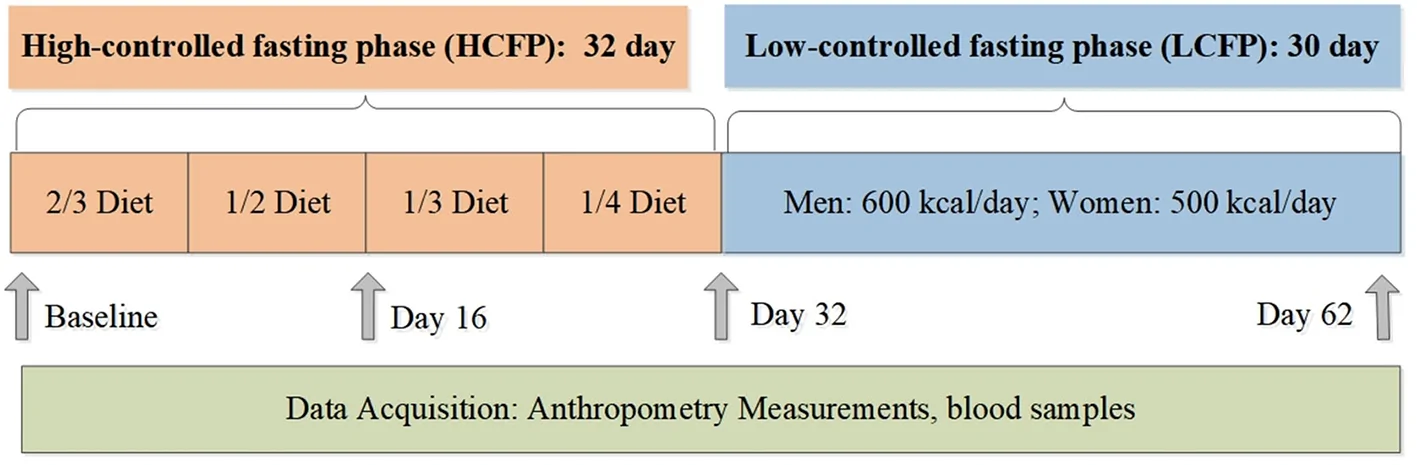
Schematic overview of the study design. Participants underwent a 62-day ADMF protocol consisting of a 32-day HCFP with stepwise caloric restriction (2/3, 1/2, 1/3, and 1/4 of baseline daily intake over four 8-day stages) and a 30-day LCFP with 500–600 kcal/day on fasting days. Anthropometry measurements and blood samples were collected at Baseline (Day 0), Day 16, Day 32, and Day 62. ADMF, alternate-day modified fasting; HCFP, high-controlled fasting phase; LCFP, low-controlled fasting phase.

This was a 62-day single-arm trial designed to explore the impact of ADMF on body composition and cardiometabolic indices in individuals with obesity in China, which has been comprehensively detailed in previous literature (15, 16). The protocol was illustrated in Figure 2. The trial consisted of two phases: (1) a 32-day high-controlled fasting phase (HCFP) and (2) a 30-day low-controlled fasting phase (LCFP). All participants (*n* = 35) were required to report to the research center at four time points: baseline, day 16 (16 days after initiation of HCFP), day 32 (completion of HCFP), and day 62 (after completion of the entire 62-day protocol). Before starting the ADMF intervention, a 24 h dietary survey was conducted on each participant for 4 consecutive days, with the average value serving as the reference for their daily caloric intake.

### HCFP protocol

During this phase, the diet of the participants was managed by a trained nutritionist. To monitor compliance, participants were required to take photographs of their meals on *ad libitum* days and send them to the nutritionist via WeChat. The nutritionist then provided real-time feedback on food choices and fat content via the same platform. Weekly dietary recall interviews were also conducted to reinforce adherence to the fat restriction. Exercise recommendations, including suggested types of physical activity and recommended duration, were also provided by nutritionist; however, exercise adherence was not supervised. Weekly health education courses were also provided to ensure the compliance of participants and improve the effectiveness of health management. The health education course was conducted by three nutritionists and one health management professional, and it included the following topics: obesity risk, healthy eating patterns and healthy exercise patterns.

Participants underwent four stages of HCFP, each lasting 8 days. Both fasting and *ad libitum* food intake started at 0:00 am each day. On the fasting day, the subjects were instructed to reduce their energy intake to 2/3 (first stage, 1–8 days), 1/2 (second stage, 9–16 days), 1/3 (third stage, 17–24 days) and 1/4 (fourth stage, 25–32 days) of the average daily caloric intake (Figure 2). The graded restriction model was adopted to improve tolerance and adherence by allowing gradual adaptation. Food on the fasting day was prepared and distributed by the Clinical Nutrition Department of Henan Provincial People's Hospital and consisted of oats, low-energy lean meat, eggs, vegetables and supplements (dietary fiber and vitamins, produced by Nantong Licheng Co., Ltd.). In addition, drinking a large amount of water or tea was also encouraged, but sugary beverages were prohibited. On the *ad libitum* food intake day, the subjects could eat freely, but the consumption of a high-fat diet (≥30% of kcal from fat) was limited.

### LCFP protocol

This phase was conducted after the HCFP and lasted for 30 days. Participants were still advised to adhere to an energy-restricted diet (600 kcal/day for men, 500 kcal/day for women) on the alternating day at home. Daily food was prepared by the subjects at home. After 30 days, participants were required to visit the research center again, after which outcome measurements of body composition, blood pressure (BP) and biochemical indices were obtained.

### 2.3 Anthropometric assessment

Height was measured using a wall-mounted stadiometer, with an accuracy of 0.5 cm, while the participants were standing without shoes. Waist circumference (WC) and hip circumference (HC) were measured with non-elastic flexible tape (Seca 201. Hamburg, Germany) at the highest point of the iliac crest around the abdomen and at the level of the greater trochanter, respectively, and the closest 0.1 cm was recorded. Weight and other body composition indices, such as body fat mass (BFM), fat-free mass (FFM), soft lean mass (SLM), skeletal muscle mass (SMM), percent body fat (PBF), visceral fat area (VFA) and waist-to-hip ratio (WHR), were measured by an 8-point tactile electrode bioelectrical impedance analyzer (BIA; InBody 770; InBody Co., Ltd., Seoul, Korea). Measurements were conducted under standardized conditions in accordance with the manufacturer's protocol to ensure accurate measurement values: bowels and bladder emptied, fasted in the morning, wore light clothes, and rested for 5 min.

### 2.4 Blood pressure (BP)

BP was measured by a corrected electronic sphygmomanometer while the participants were in the seated position after a 15 min rest in the morning between 7:00 and 9:00. According to the National High Blood Pressure Education Working Group recommendations (14), the average of three consecutive measurements for systolic blood pressure (SBP) and diastolic blood pressure (DBP) was used for analysis.

### 2.5 Biochemical indicator collection

Peripheral venous blood samples were collected after 12 h of overnight fasting. The blood samples were centrifuged at 3500 rpm for 15 min to obtain serum samples, after which the levels were measured within 4 h. The total cholesterol (TC), triglyceride (TG), high-density lipoprotein cholesterol (HDL-C), low-density lipoprotein (LDL-C), serum uric acid (UA), hemoglobin A1c (HbA1c) and fasting plasma glucose (FPG) levels were measured using an automatic biochemical analyzer (Beckman Cooler, Inc., Brea, CA, USA). UA levels were measured using the enzymatic colorimetric method, and FPG levels were measured using the hexokinase/glucose-6-phosphate dehydrogenase method.

### 2.6 Statistical analysis

For the sample size calculation, given the exploratory nature of the single-arm study, which was designed to generate preliminary evidence rather than test a confirmatory hypothesis, a formal *a priori* power calculation was not performed. The sample size was determined with reference to a previous clinical study using a similar ADMF protocol (*n* = 16) (17). Considering the potential for a 20% dropout rate during the 62-day intervention, we planned to recruit at least 20 participants to ensure adequate evaluation of changes in the primary outcomes. Ultimately, 35 participants completed the trial, exceeding the minimum requirement and providing sufficient statistical power for the analysis.

All statistical analyses were conducted using IBM statistics (SPSS) V.17. Normality was evaluated through the Kolmogorov–Smirnov test. For normally distributed data, the mean ± standard deviation (M±SD) was used for results presentation, and paired *t*-test was used for comparisons between two time points. When the data distribution was abnormal, the results are presented as the median and upper and lower quartiles [M, (Q1, Q3)], and Wilcoxon signed-rank test was used for comparisons between two time points. For correlation analysis, Pearson's correlation coefficient (*r*) was used for normally distributed variables, while Spearman's rank correlation coefficient (ρ) was used for non-normally distributed variables. A *P* value of < 0.05 was considered to indicate statistical significance.

## 3 Results

### 3.1 Study characteristics

In total, 35 subjects completed the entire 62-day trial and were included in the analysis: 20 males (57.10%) and 15 females (42.90%). The ages of the respondents were between 22 and 55 years, with an average age of 36.23 ± 8.98 years. At baseline, the weight was between 72 kg and 137 kg, and the mean weight was 100.02 ± 16.89 kg, mean BMI was 35.47 ± 4.15kg/m^2^. The baseline characteristics of the study participants are presented in Table 1.

**Table 1:** Anthropometric, blood pressure, and biochemical parameters at baseline and during the intervention.

					Changes in HCFP			Changes in LCFP			Changes in ADMF		
Variables	Baseline	Day16	Day32	Day62	Changes	%	*P_*HCFP*_*	Changes	%	*P_*LCFP*_*	Changes	%	*P_*ADMF*_*
BW (kg)	100.02 ± 16.89	95.98 ± 16.61	93.66 ± 16.50	92.14 ± 15.99	−6.29 ± 3.11	−6.38 ± 3.09	**< 0.001**	−0.32 ± 2.50	−0.47 ± 2.88	0.463	−6.83 ± 4.26	−7.02 ± 4.53	**< 0.001**
BFM (kg)	40.95 ± 9.35	38.16 ± 9.04	36.37 ± 9.35	35.13 ± 9.37	−4.43 ± 2.36	−11.14 ± 5.80	**< 0.001**	−0.83 ± 1.73	−2.45 ± 5.07	**0.008**	−5.38 ± 3.13	−13.61 ± 7.57	**< 0.001**
SLM (kg)	55.81 ± 9.80	54.58 ± 9.91	54.09 ± 9.46	53.81 ± 9.08	−1.80 ± 1.57	−3.40 ± 2.92	**< 0.001**	0.46 ± 1.19	0.73 ± 2.42	**0.031**	−1.44 ± 1.88	−2.68 ± 3.59	**< 0.001**
FFM (kg)	59.07 ± 10.47	57.82 ± 10.58	57.29 ± 10.08	57.01 ± 9.64	−1.86 ± 1.68	−3.32 ± 2.95	**< 0.001**	0.51 ± 1.25	0.78 ± 2.40	**0.022**	−1.45 ± 1.99	−2.54 ± 3.55	**< 0.001**
SMM (kg)	33.14 ± 6.27	32.44 ± 6.30	32.03 ± 6.03	31.79 ± 5.74	−1.16 ± 1.00	−3.73 ± 3.12	**< 0.001**	0.23 ± 0.71	0.58 ± 2.45	0.071	−0.98 ± 1.16	−3.09 ± 3.74	**< 0.001**
BMI (kg/m^2^)	35.47 ± 4.15	33.93 ± 4.05	33.12 ± 4.14	32.66 ± 3.83	−2.28 ± 1.19	−7.02 ± 3.67	**< 0.001**	−0.17 ± 0.98	−0.49 ± 3.01	0.315	−2.53 ± 1.62	−7.13 ± 4.38	**< 0.001**
PBF (%)	40.82 ± 5.16	39.64 ± 5.36	38.62 ± 5.42	37.87 ± 5.55	−2.06 ± 1.58	−5.62 ± 4.56	**< 0.001**	−0.79 ± 1.18	−2.05 ± 2.98	**< 0.001**	−2.90 ± 1.86	−7.23 ± 5.22	**< 0.001**
WHR (%)	1.01 ± 0.07	1.00 ± 0.07	0.99 ± 0.07	0.98 ± 0.07	−0.02 ± 0.01	−2.29 ± 1.50	**< 0.001**	−0.01 ± 0.03	−0.54 ± 2.63	0.226	−0.03 ± 0.03	−2.78 ± 2.60	**< 0.001**
VFA (cm^2^)	190.04 ± 39.54	177.65 ± 40.69	169.37 ± 42.99	164.44 ± 43.84	−19.97 ± 10.67	−13.10 ± 8.46	**< 0.001**	−4.53 ± 8.96	−2.80 ± 5.61	**0.006**	−24.98 ± 13.77	−13.89 ± 8.07	**< 0.001**
WC (cm)	114.67 ± 12.13	111.32 ± 12.12	108.93 ± 12.43	107.77 ± 12.74	−5.68 ± 2.67	−5.35 ± 2.59	**< 0.001**	−1.11 ± 3.03	−1.02 ± 2.75	**0.044**	−6.94 ± 4.31	−6.11 ± 3.81	**< 0.001**
HC (cm)	113.54 ± 7.16	111.51 ± 7.09	110.15 ± 7.26	109.30 ± 6.49	−3.30 ± 1.88	−3.03 ± 1.73	**< 0.001**	−0.35 ± 1.56	−0.33 ± 1.43	0.204	−3.79 ± 2.47	−3.35 ± 2.18	**< 0.001**
SBP (mmHg)	129.45 ± 15.65	124.00 ± 12.10	123.26 ± 12.66	122.22 ± 13.86	−7.10 ± 14.94	−4.65 ± 11.22	**0.013**	−1.13 ± 12.55	−0.46 ± 10.20	0.616	−9.28 ± 11.28	−6.63 ± 8.43	**< 0.001**
DBP (mmHg)	78.42 ± 10.34	75.56 ± 11.01	74.03 ± 10.30	74.91 ± 11.77	−5.52 ± 9.42	−6.24 ± 11.65	**0.003**	1.34 ± 8.68	2.19 ± 11.08	0.388	**–**4.66 ± 8.85	−5.37 ± 11.25	**0.008**
UA (μmol/L)	402.12 ± 112.57	463.39 ± 118.31	432.68 ± 111.57	391.68 ± 110.30	27.30 ± 79.60	8.85 ± 11.18	**0.047**	−33.70 ± 72.48	−6.67 ± 15.88	**0.012**	−8.61 ± 84.36	**–**0.20 ± 21.87	0.562
TC (mmol/L)	4.78 ± 0.99	4.66 ± 1.03	4.63 ± 0.88	4.77 ± 0.84	−0.18 ± 0.75	−4.71 (−11.12, 4.93)	0.180	0.15 ± 0.62	4.25 ± 14.12	0.184	0.00 ± 0.60	1.39 ± 12.30	0.989
TG (mmol/L)	2.12 (1.38, 3.14)	1.53 (1.33, 2.15)	1.81 (1.35, 2.58)	1.78 (1.39, 2.39)	−0.24 ± 0.82	−4.92 ± 28.20	0.097	0.02 (−0.13, 0.58)	11.97 ± 38.77	0.417	−0.09 ± 1.00	1.50 ± 37.64	0.599
HDL (mmol/L)	1.11 ± 0.16	1.02 ± 0.13	1.04 ± 0.15	1.03 ± 0.15	−0.07 ± 0.14	−5.10 ± 12.74	**0.009**	−0.01 (−0.07, 0.04)	1.72 (−6.83, 40.42)	0.925	−0.07 ± 0.15	−5.24 ± 13.71	**0.015**
LDL (mmol/L)	2.71 ± 0.77	2.69 ± 0.81	2.63 ± 0.70	2.67 ± 0.72	−0.10 ± 0.52	−1.31 ± 19.87	0.270	0.04 ± 0.49	3.36 ± 20.50	0.632	−0.03 ± 0.41	0.41 ± 15.83	0.641
FPG (mmol/L)	4.91 ± 0.95	4.33 ± 0.45	4.38 ± 0.72	4.58 ± 0.62	−0.52 ± 0.89	−9.48 ± 12.78	**0.002**	0.21 ± 0.63	6.09 ± 13.57	0.062	−0.33 ± 0.76	−5.35 ± 11.97	**0.018**

Values are presented as mean ± SD for normally distributed variables and as median (Q1, Q3) for non-normally distributed variables. *P*_HCFP_, changes from Baseline to Day 32; *P*__*L*_CFP_, changes from Day 32 to Day 62; *P*_ADMF_, changes from Baseline to Day 62 (overall ADMF effect). BW, body weight; BFM, body fat mass; SLM, soft lean mass; FFM, fat-free mass; SMM, skeletal muscle mass; BMI, body mass index; PBF, percent body fat; WHR, waist-to-hip ratio; VFA, visceral fat area; WC, waist circumference; HC, hip circumference. SBP, systolic blood pressure; DBP, diastolic blood pressure; UA, uric acid; TC, total cholesterol; TG, triglyceride; HDL, high-density lipoprotein cholesterol; LDL, low-density lipoprotein; FPG, fasting plasma glucose. Bold values indicate statistical significance (*P* < 0.05).

### 3.2 Body weight and body composition

The changes in the body composition characteristics of the subjects at different time points of intervention are presented in Table 1. Over the entire 62-day ADMF intervention, body weight (BW) decreased by −6.83 ± 4.26 kg (−7.02%, *P* < 0.001), accompanied by significant reductions in BFM (−5.38 ± 3.13 kg, −13.61%, *P* < 0.001), FFM (-1.45±1.99 kg, −2.54%), VFA (−24.98 ± 13.77 cm^2^, −13.89%, *P* < 0.001), and WC (−6.94 ± 4.31 cm, −6.11%, *P* < 0.001), while SLM (−1.44 ± 1.88 kg, −2.68%, *P* < 0.001) and SMM (−0.98 ± 1.16 kg, −3.09%, *P* < 0.001) also decreased significantly. During HCFP, significant decreases from baseline were observed for weight (−6.38%), BFM (−11.14%), SLM (−3.40%), FFM (−3.32%), SMM (−3.73%), BMI (−7.02%), PBF (−5.62%), WHR (−2.29%), VFA (−13.10%), WC (−5.35%), and HC (−3.03%), all with statistical significance (*P* < 0.001). During LCFP, significant decreases were observed in BFM (−2.45%), PBF (−2.05%), VFA (−2.80%), and WC (−1.02%), as well as significant increases in SLM (0.73%) and FFM (0.78%), all with statistical significance (*P* < 0.05).

**Figure 3:**
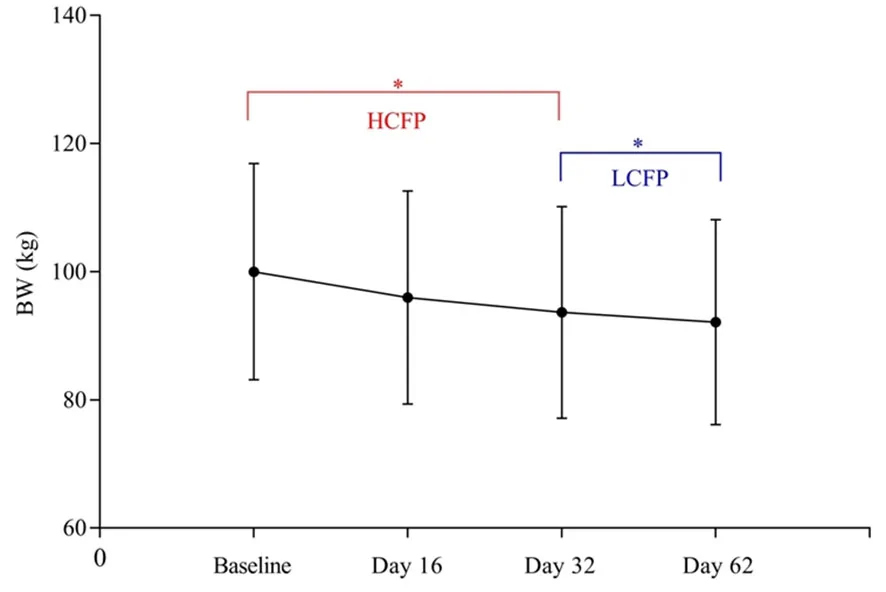
Longitudinal changes in body weight across the 62-day intervention. Data are presented as mean ± SD at four timepoints: baseline, Day 16 (midpoint of HCFP), Day 32 (endpoint of HCFP), and Day 62 (endpoint of LCFP). *Significant difference (*P* < 0.05) compared with the changes during HCFP (paired sample *t* test). BW, body weight; SD, standard deviation; HCFP, high-controlled fasting phase; LCFP, low-controlled fasting phase.

**Figure 4:**
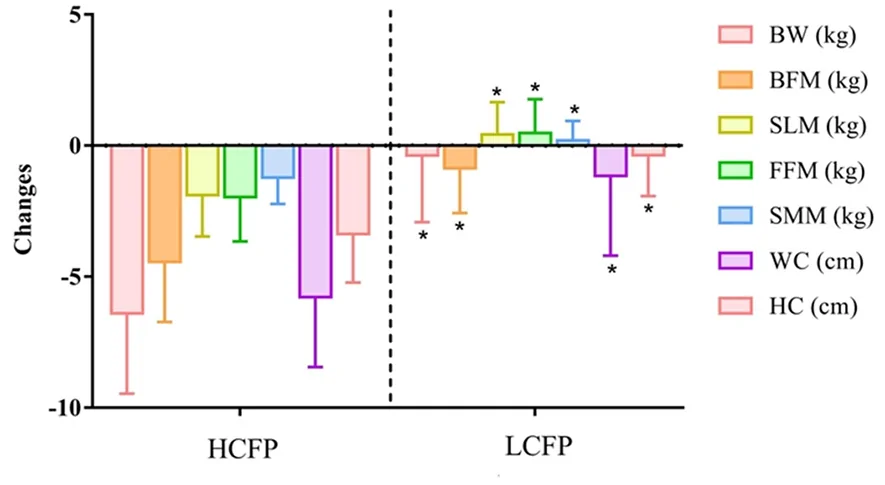
Changes in weight and body composition during the two periods. Values are reported as the mean ± SD. *Significant difference (*P* < 0.05) compared with the changes during HCFP (paired sample *t* test). Values are reported as the mean ± SD. BW, body weight; BFM, body fat mass; SLM, soft lean mass; FFM, fat-free mass; SMM, skeletal muscle mass; WC, waist circumference; HC, hip circumference; SD, standard deviation; HCFP, high-controlled fasting phase; LCFP, low-controlled fasting phase.

The longitudinal trajectory of BW across the four timepoints is illustrated in Figure 3, which shows a progressive decline during HCFP (from 100.02 ± 16.89 kg at baseline to 93.66 ± 16.50 kg at Day 32), followed by a slower decline during LCFP (to 92.14 ± 15.99 kg at Day 62), with significant differences (*P* < 0.05). The proportion of total weight loss attributable to FFM was 22.68%. The comparison of changes in body composition between HCFP and LCFP is shown in Figure 4. The changes in body composition indicators during HCFP were significantly higher than those in LCFP (*P* < 0.05).

### 3.3 Blood pressure and metabolic-related indicators

**Figure 5:**
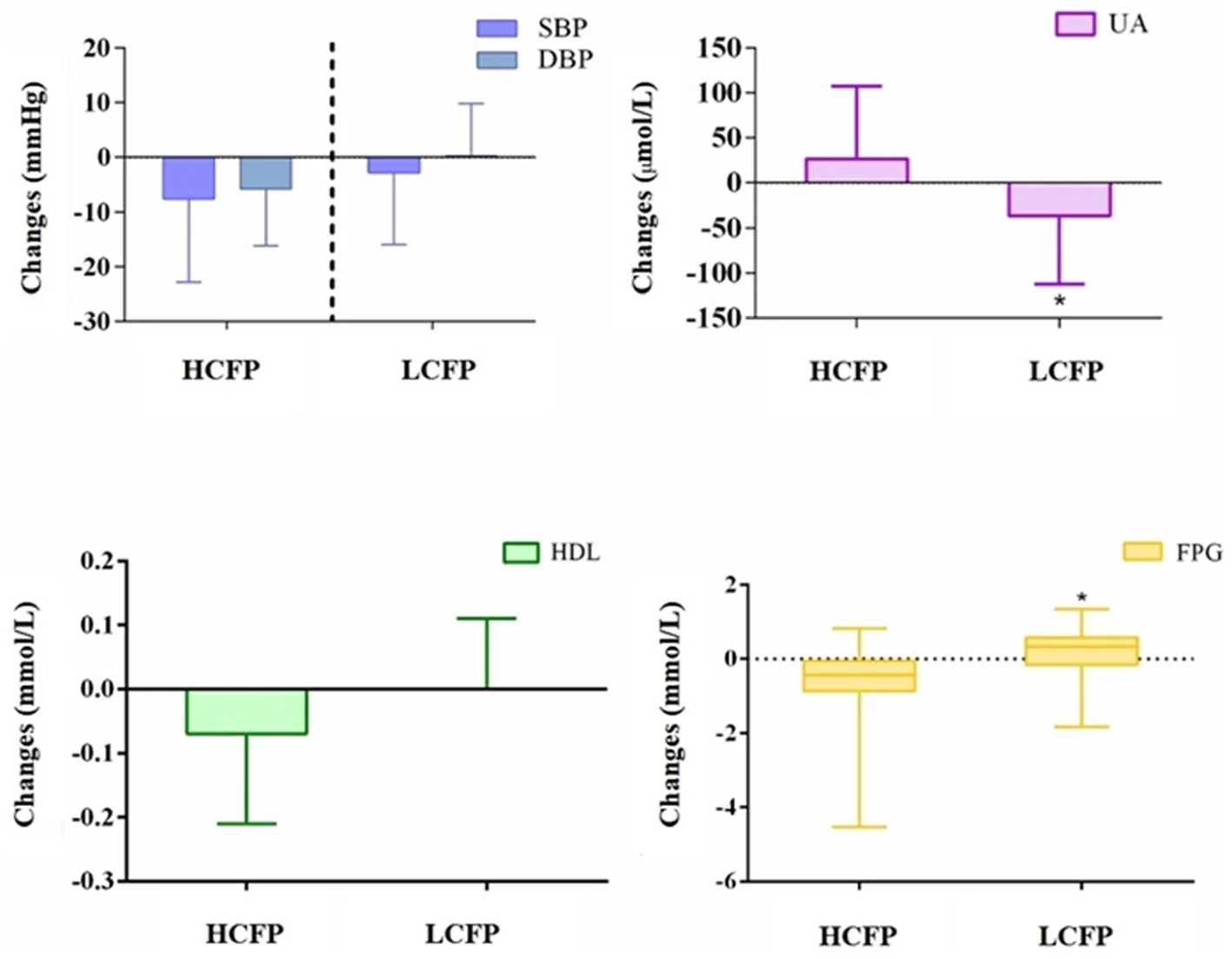
Changes in blood pressure and metabolic indices during the two periods. Values are reported as the mean ± SD. *Significant difference (*P* < 0.05) compared with the changes during HCFP (paired sample *t* test or rank-sum test). Values are reported as the mean ± SD or median (1st−3rd quartile). SBP, systolic blood pressure; DBP, diastolic blood pressure; UA, uric acid; HDL, high-density lipoprotein cholesterol; FPG, fasting plasma glucose; SD, standard deviation; HCFP, high-controlled fasting phase; LCFP, low-controlled fasting phase.

The changes in the BP values and biochemical parameters are presented in Table 1. Over the entire 62-day ADMF intervention, SBP (−9.28 ± 11.28 mmHg, −6.63%, *P* < 0.001), DBP (−4.66 ± 8.85 mmHg, −5.37%, *P* = 0.008), FPG (−0.33 ± 0.76 mmol/L, −5.35%, *P* = 0.010), and HDL (−0.07 ± 0.15 mmol/L, −5.24%, *P* = 0.015) decreased significantly, whereas TC, TG, LDL, and UA showed no significant changes (all *P* > 0.05). During HCFP, significant changes were observed for SBP (−4.65%), DBP (−6.24%), UA (8.85%), HDL (−5.01%) and FPG (−9.48%), all with statistical significance (*P* < 0.05). During LCFP, significant change was only observed in UA (−6.67%). The comparison of changes in BP values and biochemical parameters between HCFP and LCFP is shown in Figure 5. The changes of UA and FPG were significantly different during HCFP and LCFP (*P* < 0.05). It is worth noting that UA levels significant elevated during HCFP, but significant decreased during LCFP.

### 3.4 Analysis of factors related to UA

Before and after the intervention, correlation analysis demonstrated that UA was positively correlated with weight, SLM, FFM and SMM. Furthermore, after adjusting for age and gender, the results remained consistent (*P* < 0.05, Tables 2, 3).

**Table 2:** Correlations between baseline UA and other baseline parameters.

Variables	Baseline	UA (μmol/L)		Adjustment for age and sex	
		***r/***ρ	***P***	***r/***ρ	***P***
BW (kg)	100.02 ± 16.89	0.387	0.024	0.301	**0.044**
BFM (kg)	40.95 ± 9.35	0.121	0.494	0.061	0.664
SLM (kg)	55.81 ± 9.80	0.515	0.002	0.415	**0.012**
FFM (kg)	59.07 ± 10.47	0.516	0.002	0.340	**0.035**
SMM (kg)	33.14 ± 6.27	0.525	0.001	0.316	**0.040**
BMI (kg/m^2^)	35.47 ± 4.15	0.057	0.749	0.065	0.639
PBF (%)	40.82 ± 5.16	−0.294	0.088	−0.017	0.918
WHR (%)	1.01 ± 0.07	0.166	0.347	−0.101	0.504
VFA (cm^2^)	190.04 ± 39.54	0.017	0.926	0.012	0.993
WC (cm)	114.67 ± 12.13	0.275	0.116	0.038	0.803
HC (cm)	113.54 ± 7.16	0.291	0.095	0.148	0.295
SBP (mmHg)	129.45 ± 15.65	0.170	0.360	0.113	0.439
DBP (mmHg)	78.42 ± 10.34	0.517	0.002	0.187	0.237
TC (mmol/L)	4.78 ± 0.99	0.102	0.566	0.151	0.287
TG (mmol/L)^†^	2.12 (1.38, 3.14)	0.383	0.025	0.083	0.576
HDL (mmol/L)	1.11 ± 0.16	−0.084	0.636	0.162	0.301
LDL (mmol/L)	2.71 ± 0.77	0.044	0.805	0.139	0.318
FPG (mmol/L)	4.91 ± 0.95	0.081	0.650	−0.099	0.490

Values are presented as mean ± SD for normally distributed variables and as median (Q1, Q3) for non-normally distributed variables. For normally distributed variables, Pearson correlation coefficient (*r*) was used; for non-normally distributed variables (TG), Spearman rank correlation coefficient (ρ) was used for non-normally distributed variables (marked with †). BW, body weight; BFM, body fat mass; SLM, soft lean mass; FFM, fat-free mass; SMM, skeletal muscle mass; BMI, body mass index; PBF, percent body fat; WHR, waist-to-hip ratio; VFA, visceral fat area; WC, waist circumference; HC, hip circumference. SBP, systolic blood pressure; DBP, diastolic blood pressure; UA, uric acid; TC, total cholesterol; TG, triglyceride; HDL, high-density lipoprotein cholesterol; LDL, low-density lipoprotein; FPG, fasting plasma glucose. Bold values indicate statistical significance (*P* < 0.05).

**Table 3:** Correlations between UA and other parameters at Day 62.

Variables	62	UA (μmol/L)		Adjustment for age and sex	
		***r/***ρ	***P***	***r/***ρ	***P***
BW (kg)	92.14 ± 15.99	0.397	0.018	0.284	**0.044**
BFM (kg)	35.13 ± 9.37	0.178	0.305	0.061	0.664
SLM (kg)	53.81 ± 9.08	0.485	0.000	0.415	**0.012**
FFM (kg)	57.01 ± 9.64	0.481	0.000	0.340	**0.035**
SMM (kg)	31.79 ± 5.74	0.491	0.003	0.316	**0.040**
BMI (kg/m^2^)	32.66 ± 3.83	0.135	0.438	0.065	0.639
PBF (%)	37.87 ± 5.55	−0.095	0.588	−0.017	0.918
WHR (%)	0.98 ± 0.07	0.289	0.092	−0.101	0.504
VFA (cm^2^)	164.44 ± 43.84	0.112	0.521	0.012	0.993
WC (cm)	107.77 ± 12.74	0.338	0.050	0.038	0.803
HC (cm)	109.30 ± 6.49	0.340	0.049	0.148	0.295
SBP (mmHg)	122.22 ± 13.86	0.285	0.114	0.059	0.685
DBP (mmHg)	74.91 ± 11.77	0.238	0.190	0.047	0.750
TC (mmol/L)	4.77 ± 0.84	0.069	0.697	0.086	0.496
TG (mmol/L)^†^	1.78(1.39, 2.39)	0.322	0.064	0.025	0.844
HDL (mmol/L)	1.03 ± 0.15	−0.165	0.352	−0.066	0.620
LDL (mmol/L)	2.67 ± 0.71	0.083	0.640	0.098	0.433
FPG (mmol/L)	4.58 ± 0.62	0.085	0.633	−0.009	0.945

Values are presented as mean ± SD for normally distributed variables and as median (Q1, Q3) for non-normally distributed variables. For normally distributed variables, Pearson correlation coefficient (*r*) was used; for non-normally distributed variables (TG), spearman rank correlation coefficient (ρ) was used (marked with †). BW, body weight; BFM, body fat mass; SLM, soft lean mass; FFM, fat-free mass; SMM, skeletal muscle mass; BMI, body mass index; PBF, percent body fat; WHR, waist-to-hip ratio; VFA, visceral fat area; WC, waist circumference; HC, hip circumference. SBP, systolic blood pressure; DBP, diastolic blood pressure; UA, uric acid; TC, total cholesterol; TG, triglyceride; HDL, high-density lipoprotein cholesterol; LDL, low-density lipoprotein; FPG, fasting plasma glucose. Bold values indicate statistical significance (*P* < 0.05).

As for the changes, the correlation analysis indicated that the change of UA was negatively correlated with the relative change of weight, SLM, WC during the first 16 days. By contrast, during LCFP, the change of UA was positively correlated with the changes of BFM, PBF, WHR, VFA and WC (*P* < 0.05, Table 4).

**Table 4:** Correlations between changes in UA and changes in body composition parameters across different phases.

Variables	Relative changes (Day 16–Baseline)	UA (μmol/L)		Relative changes (Day 32–Day 16)	UA (μmol/L)		Relative changes (Day 62–Day 32)	UA (μmol/L)	
		***r***	***P***		***r***	***P***		***r***	***P***
BW (kg)	−3.97 ± 1.83	**−0.410**	**0.015**	−2.32 ± 1.85	0.241	0.822	−0.32 ± 2.50	0.293	0.099
BFM (kg)	−2.63 ± 1.39	−0.046	0.802	−1.79 ± 1.49	0.253	0.163	−0.83 ± 1.73	**0.389**	**0.025**
SLM (kg)	−1.31 ± 1.16	**−0.340**	**0.025**	−0.49 ± 1.19	−0.224	0.218	0.46 ± 1.19	0.003	0.987
FFM (kg)	−1.34 ± 1.24	−0.324	0.062	−0.53 ± 1.27	−0.217	0.233	0.51 ± 1.25	−0.010	0.955
SMM (kg)	−0.76 ± 0.73	−0.267	0.139	−0.40 ± 0.70	−0.122	0.505	0.23 ± 0.71	0.066	0.716
BMI (kg/m^2^)	−1.47 ± 0.69	−0.025	0.892	−0.81 ± 0.72	0.053	0.773	−0.17 ± 0.98	0.240	0.179
PBF (%)	−1.04 ± 0.97	0.028	0.877	−1.03 ± 1.22	0.341	0.056	−0.79 ± 1.18	**0.408**	**0.018**
WHR (%)	−0.01 ± 0.02	−0.295	0.101	−0.01 ± 0.02	0.092	0.616	−0.01 ± 0.03	**0.407**	**0.019**
VFA (cm^2^)	−11.71 ± 6.29	−0.164	0.369	−8.28 ± 7.87	0.218	0.231	−4.53 ± 8.96	**0.492**	**0.004**
WC (cm)	−3.26 ± 1.83	**−0.376**	**0.034**	−2.40 ± 2.09	0.194	0.287	−1.11 ± 3.03	**0.499**	**0.004**
HC (cm)	−3.06 ± 7.57	−0.122	0.504	−1.36 ± 1.13	0.190	0.298	−0.35 ± 1.56	0.180	0.324

Values are reported as the mean ± SD. BW, body weight; BFM, body fat mass; SLM, soft lean mass; FFM, fat-free mass; SMM, skeletal muscle mass; BMI, body mass index; PBF, percent body fat; WHR, waist-to-hip ratio; UA, uric acid; VFA, visceral fat area; WC, waist circumference; HC, hip circumference. Bold values indicate statistical significance (*P* < 0.05).

## 4 Discussion

This study demonstrated that a structured two-phase ADMF intervention (including a 32-day HCFP and a 30-day LCFP) was associated with reduced body weight and adiposity while improving certain cardiometabolic risk factors in Chinese adults with obesity. The most salient findings, however, revealed a dual phase-dependent effects: the intensive HCFP induced significant loss of lean body mass and a transient rise in UA, whereas the subsequent LCFP was associated with partial lean mass recovery and the normalization of UA levels. To our knowledge, this is the first study to systematically characterize the temporal dynamics of UA in conjunction with detailed body composition changes during an ADMF protocol in individuals with obesity.

Our results were consistent with previous findings regarding the efficacy of ADMF for reducing overall and visceral adiposity. The significant decrease in body weight (−6.38%), BFM (−11.14%), WC (−5.35), and particularly VFA (−13.10) during the HCFP, which align with previous researches suggesting ADMF as an effective weight-loss strategy (9, 18, 19). The continued reduction in these adiposity indices during LCFP was maintained with a lower intervention intensity, which might improve long-term adherence. This is encouraging, suggesting that some metabolic benefits could be maintained. The reduction in VFA is of paramount importance, given its strong causal link to insulin resistance and cardiometabolic disease (20, 21).

However, the significant decrease in SLM, FFM, and SMM observed during the HCFP warrants careful consideration. Preserving lean mass is critical during weight loss, as it is the primary determinant of resting metabolic rate and essential for physical function. The muscle loss may undermine long-term weight maintenance and predispose individuals to sarcopenic obesity (22). The greater changes observed during HCFP compared with LCFP can be attributed to the substantially larger actual cumulative energy deficit experienced during the highly controlled phase, which involved stepwise caloric restriction down to one-quarter of baseline intake with full dietary supervision. In contrast, although the prescribed 500–600 kcal/day during LCFP was theoretically comparable to the final HCFP stage, this phase relied on unsupervised home-based adherence, which likely resulted in reduced compliance and attenuated metabolic changes. The significant recovery of SLM and FFM during the LCFP suggested that the catabolic state driven by severe energy restriction was alleviated when dietary control was relaxed. This pattern underscores that the metabolic adaptations to ADMF were not static but varied with the intensity of the intervention. It further implied that strategies to mitigate muscle loss, such as ensuring adequate protein intake and incorporating resistance training, should be integral components of intensive ADMF phases (23, 24).

The most notable finding was the biphasic change in UA levels with a significant increase during the HCFP followed by a significant decrease during LCFP. This pattern likely reflects the interplay of renal handling and purine metabolism under varying dietary stresses. The initial UA elevation during HCFP may be attributed to two concurrent mechanisms. First, severe carbohydrate restriction promotes ketogenesis, where ketone bodies (primarily β-hydroxybutyrate) compete with UA for renal excretion via organic anion transporters in the proximal tubule, temporarily reducing UA clearance (25, 26). Second, the significant loss of SLM (−3.40%) and SMM (−3.73%) suggests increased proteolysis and muscle turnover, which may augment the endogenous purine pool available for conversion to UA (27). Our observation of a negative correlation between changes in UA and SLM during the HCFP (*r* = −0.340, *P* = 0.025) supports this association, consistent with evidence that lower lean mass is linked to hyperuricemia (28, 29). However, this correlation does not establish causality, as concurrent factors such as hydration status, dietary protein intake, and acute changes in renal hemodynamics may also contribute. Additionally, the graded caloric restriction protocol may confound the effects of time with those of escalating energy deficit, future studies comparing graded vs. fixed restriction regimens are warranted to isolate the independent contributions of duration vs. intensity.

Conversely, the decrease in UA during the LCFP (−6.67%) likely reflects a reversal of these processes. The increased of carbohydrates intake would have reduced ketone-mediated competition for renal excretion, while the partial recovery of SLM (+0.73%) and continued fat loss would have improved insulin sensitivity, facilitating enhanced renal UA clearance (9, 10, 15). While no gout events occurred in our short-term trial, elevated UA is an established independent risk factor for cardiovascular disease and the fundamental precursor for gout (25). Although the clinical relevance of this transient UA elevation remains to be determined, monitoring UA levels may be considered on an individualized basis, particularly for individuals with pre-existing hyperuricemia, impaired renal function, or a history of gout.

Several limitations of this study must be acknowledged. First, the 62-day duration, while sufficient to capture acute metabolic shifts, was too short to evaluate long-term sustainability, safety (particularly regarding gout risk), and health outcomes. Second, the lack of detailed dietary records on *ad libitum* intake days limited our ability to analyze total energy and macronutrient intake, which could provide further mechanistic insights. Third, body composition was assessed using BIA, the accuracy of which is susceptible to factors such as hydration status. Therefore, the observed changes in body composition should be interpreted with caution. In addition, body weight was measured at only four key timepoints, more frequent monitoring would have enabled a more detailed kinetic assessment. Future studies incorporating imaging modalities such as CT or MRI are warranted to further validate the findings of this study. Forth, our sample was recruited from a single center, and a larger, multicenter randomized controlled trials are needed to confirm these findings and enhance their generalizability. Fifth, the graded caloric restriction protocol may introduce confounding between time and restriction severity; thus, the progressive changes in UA and body composition should be interpreted with caution, and future studies comparing graded with fixed restriction protocols are warranted. Finally, as a single-arm study without a control group, we cannot definitively attribute all observed changes to the ADMF intervention. The results should therefore be interpreted as exploratory findings that generate hypotheses rather than confirmatory evidence of causality, and warrant validation in larger, randomized controlled trials.

## 5 Conclusions

In summary, this two-phase ADMF protocol may be effective for reducing obesity and improving several cardiometabolic parameters in Chinese adults with obesity. Its effects, however, were phase-dependent. The intensive HCFP drove rapid fat loss but was associated with decreases in SLM and SMM as estimated by BIA, and a transient hyperuricemia. The subsequent LCFP slowed fat reduction, but allowed for partial increases in estimated lean mass parameters, and decreased UA levels. These findings advocate for a nuanced application of ADMF. Future implementations should consider a phased approach, explicitly differentiating between intensive weight-loss and moderate maintenance phases. Optimizing such protocols with muscle-preserving strategies (e.g., high-protein diets, resistance exercise) may be considered, and UA monitoring could be considered on an individualized basis, particularly for individuals with pre-existing hyperuricemia or a history of gout. The clinical relevance of the transient UA elevation remains to be determined in future long-term studies.

## Data Availability

The raw data supporting the conclusions of this article will be made available by the corresponding author upon request.
