# Supplementary material for: Excessive weight loss during alternate day modified fasting increases the risk of hyperuricemia and muscle loss
Source: Front Nutr. 2026 Sep 9;13:1842818. doi: 10.3389/fnut.2026.1842818 (PMC13597861; doi:10.3389/fnut.2026.1842818)
Supplement: Supplementary file 1 [file Supplementary_File_1.DOCX]

Supplementary Material

Excessive Weight Loss during Alternate Day Modified Fasting Increases the Risk of Hyperuricemia and Muscle Loss

Yanjuan Zhao^1*^, Tingting Yang^1*^, Wen Wang^1*^, Jing Wen^1^, Yan Qiao^2^, Zhi Zou^3^, Jing Zhou^4^, Xiaoling Wu^5^, Yongchun Chen^1#^, Yongli Li^3#^

**^#^Correspondence:** Yongli Li, E-mail, [shyliyongli@126.com](mailto:shyliyongli@126.com) and Yongchun Chen, E-mail: [841874197@qq.com](mailto:841874197@qq.com)

**Number of tables:** 4

**Number of figures:**5

Table 1 Anthropometric, blood pressure, and biochemical parameters at baseline and during the intervention

| Variables | Baseline | Day 16 | Day 32 | Day 62 | Changes in HCFP | | *P_HCFP_* | Changes in LCFP | | *P_LCFP_* | Changes in ADMF | | *P_ADMF_* |
| --- | --- | --- | --- | --- | --- | --- | --- | --- | --- | --- | --- | --- | --- |
|  |  |  |  |  | changes | % |  | changes | % |  | changes | % |  |
| BW(kg) | 100.02±16.89 | 95.98±16.61 | 93.66±16.50 | 92.14±15.99 | -6.29±3.11 | -6.38±3.09 | **<0.001** | -0.32±2.50 | -0.47±2.88 | 0.463 | -6.83±4.26 | -7.02±4.53 | **<0.001** |
| BFM(kg) | 40.95±9.35 | 38.16±9.04 | 36.37±9.35 | 35.13±9.37 | -4.43±2.36 | -11.14±5.80 | **<0.001** | -0.83±1.73 | -2.45±5.07 | **0.008** | -5.38±3.13 | -13.61±7.57 | **<0.001** |
| SLM(kg) | 55.81±9.80 | 54.58±9.91 | 54.09±9.46 | 53.81±9.08 | -1.80±1.57 | -3.40±2.92 | **<0.001** | 0.46±1.19 | 0.73±2.42 | **0.031** | -1.44±1.88 | -2.68±3.59 | **<0.001** |
| FFM(kg) | 59.07±10.47 | 57.82±10.58 | 57.29±10.08 | 57.01±9.64 | -1.86±1.68 | -3.32±2.95 | **<0.001** | 0.51±1.25 | 0.78±2.40 | **0.022** | -1.45±1.99 | -2.54±3.55 | **<0.001** |
| SMM(kg) | 33.14±6.27 | 32.44±6.30 | 32.03±6.03 | 31.79±5.74 | -1.16±1.00 | -3.73±3.12 | **<0.001** | 0.23±0.71 | 0.58±2.45 | 0.071 | -0.98±1.16 | -3.09±3.74 | **<0.001** |
| BMI(kg/m^2^) | 35.47±4.15 | 33.93±4.05 | 33.12±4.14 | 32.66±3.83 | -2.28±1.19 | -7.02±3.67 | **<0.001** | -0.17±0.98 | -0.49±3.01 | 0.315 | -2.53±1.62 | -7.13±4.38 | **<0.001** |
| PBF(%) | 40.82±5.16 | 39.64±5.36 | 38.62±5.42 | 37.87±5.55 | -2.06±1.58 | -5.62±4.56 | **<0.001** | -0.79±1.18 | -2.05±2.98 | **<0.001** | -2.90±1.86 | -7.23±5.22 | **<0.001** |
| WHR(%) | 1.01±0.07 | 1.00±0.07 | 0.99±0.07 | 0.98±0.07 | -0.02±0.01 | -2.29±1.50 | **<0.001** | -0.01±0.03 | -0.54±2.63 | 0.226 | -0.03±0.03 | -2.78±2.60 | **<0.001** |
| VFA(cm^2^) | 190.04±39.54 | 177.65±40.69 | 169.37±42.99 | 164.44±43.84 | -19.97±10.67 | -13.10±8.46 | **<0.001** | -4.53±8.96 | -2.80±5.61 | **0.006** | -24.98±13.77 | -13.89±8.07 | **<0.001** |
| WC(cm) | 114.67±12.13 | 111.32±12.12 | 108.93±12.43 | 107.77±12.74 | -5.68±2.67 | -5.35±2.59 | **<0.001** | -1.11±3.03 | -1.02±2.75 | **0.044** | -6.94±4.31 | -6.11±3.81 | **<0.001** |
| HC(cm) | 113.54±7.16 | 111.51±7.09 | 110.15±7.26 | 109.30±6.49 | -3.30±1.88 | -3.03±1.73 | **<0.001** | -0.35±1.56 | -0.33±1.43 | 0.204 | -3.79±2.47 | -3.35±2.18 | **<0.001** |
| SBP(mmHg) | 129.45±15.65 | 124.00±12.10 | 123.26±12.66 | 122.22±13.86 | -7.10±14.94 | -4.65±11.22 | **0.013** | -1.13±12.55 | -0.46±10.20 | 0.616 | -9.28±11.28 | -6.63±8.43 | **<0.001** |
| DBP(mmHg) | 78.42±10.34 | 75.56±11.01 | 74.03±10.30 | 74.91±11.77 | -5.52±9.42 | -6.24±11.65 | **0.003** | 1.34±8.68 | 2.19±11.08 | 0.388 | **-**4.66±8.85 | -5.37±11.25 | **0.008** |
| UA(μmol/L) | 402.12±112.57 | 463.39±118.31 | 432.68±111.57 | 391.68±110.30 | 27.30±79.60 | 8.85±11.18 | **0.047** | -33.70±72.48 | -6.67±15.88 | **0.012** | -8.61±84.36 | **-**0.20±21.87 | 0.562 |
| TC(mmol/L) | 4.78±0.99 | 4.66±1.03 | 4.63±0.88 | 4.77±0.84 | -0.18±0.75 | -4.71(-11.12,4.93) | 0.180 | 0.15±0.62 | 4.25±14.12 | 0.184 | 0.00±0.60 | 1.39±12.30 | 0.989 |
| TG(mmol/L) | 2.12(1.38, 3.14) | 1.53(1.33, 2.15) | 1.81(1.35, 2.58) | 1.78(1.39, 2.39) | -0.24±0.82 | -4.92±28.20 | 0.097 | 0.02(-0.13,0.58) | 11.97±38.77 | 0.417 | -0.09±1.00 | 1.50±37.64 | 0.599 |
| HDL(mmol/L) | 1.11±0.16 | 1.02±0.13 | 1.04±0.15 | 1.03±0.15 | -0.07±0.14 | -5.10±12.74 | **0.009** | -0.01(-0.07,0.04) | 1.72(-6.83,40.42) | 0.925 | -0.07±0.15 | -5.24±13.71 | **0.015** |
| LDL(mmol/L) | 2.71±0.77 | 2.69±0.81 | 2.63±0.70 | 2.67±0.72 | -0.10±0.52 | -1.31±19.87 | 0.270 | 0.04±0.49 | 3.36±20.50 | 0.632 | -0.03±0.41 | 0.41±15.83 | 0.641 |
| FPG(mmol/L) | 4.91±0.95 | 4.33±0.45 | 4.38±0.72 | 4.58±0.62 | -0.52±0.89 | -9.48±12.78 | **0.002** | 0.21±0.63 | 6.09±13.57 | 0.062 | -0.33±0.76 | -5.35±11.97 | **0.018** |

Values are presented as mean ± SD for normally distributed variables and as median (Q1, Q3) for non-normally distributed variables. *P*_HCFP_: changes from Baseline to Day 32; *P_L_*_CFP_:: changes from Day 32 to Day 62; *P*_ADMF_: changes from Baseline to Day 62 (overall ADMF effect). BW: body weight, BFM: body fat mass, SLM: soft lean mass, FFM: fat-free mass, SMM: skeletal muscle mass, BMI: body mass index, PBF: percent body fat, WHR: waist-to-hip ratio, VFA: visceral fat area, WC: waist circumference, HC: hip circumference. SBP: systolic blood pressure, DBP: diastolic blood pressure, UA: uric acid, TC: total cholesterol, TG: triglyceride, HDL: high-density lipoprotein cholesterol, LDL: low-density lipoprotein, FPG: fasting plasma glucose.

Table 2 Correlations between baseline UA and other baseline parameters

| Variables | Baseline | UA (μmol/L) | | Adjustment for age and sex | |
| --- | --- | --- | --- | --- | --- |
|  |  | *r/*ρ | *P* | *r/*ρ | *P* |
| BW (kg) | 100.02±16.89 | 0.387 | 0.024 | 0.301 | **0.044** |
| BFM (kg) | 40.95±9.35 | 0.121 | 0.494 | 0.061 | 0.664 |
| SLM (kg) | 55.81±9.80 | 0.515 | 0.002 | 0.415 | **0.012** |
| FFM (kg) | 59.07±10.47 | 0.516 | 0.002 | 0.340 | **0.035** |
| SMM (kg) | 33.14±6.27 | 0.525 | 0.001 | 0.316 | **0.040** |
| BMI (kg/m^2^) | 35.47±4.15 | 0.057 | 0.749 | 0.065 | 0.639 |
| PBF (%) | 40.82±5.16 | -0.294 | 0.088 | -0.017 | 0.918 |
| WHR (%) | 1.01±0.07 | 0.166 | 0.347 | -0.101 | 0.504 |
| VFA (cm^2^) | 190.04±39.54 | 0.017 | 0.926 | 0.012 | 0.993 |
| WC (cm) | 114.67±12.13 | 0.275 | 0.116 | 0.038 | 0.803 |
| HC (cm) | 113.54±7.16 | 0.291 | 0.095 | 0.148 | 0.295 |
| SBP (mmHg) | 129.45±15.65 | 0.170 | 0.360 | 0.113 | 0.439 |
| DBP (mmHg) | 78.42±10.34 | 0.517 | 0.002 | 0.187 | 0.237 |
| TC (mmol/L) | 4.78±0.99 | 0.102 | 0.566 | 0.151 | 0.287 |
| TG (mmol/L)^†^ | 2.12(1.38, 3.14) | 0.383 | 0.025 | 0.083 | 0.576 |
| HDL (mmol/L) | 1.11±0.16 | -0.084 | 0.636 | 0.162 | 0.301 |
| LDL (mmol/L) | 2.71±0.77 | 0.044 | 0.805 | 0.139 | 0.318 |
| FPG (mmol/L) | 4.91±0.95 | 0.081 | 0.650 | -0.099 | 0.490 |

Values are presented as mean ± SD for normally distributed variables and as median (Q1, Q3) for non-normally distributed variables. For normally distributed variables, pearson correlation coefficient (*r*) was used; for non-normally distributed variables (TG), Spearman rank correlation coefficient (ρ) was used for non-normally distributed variables (marked with †). BW: body weight, BFM: body fat mass, SLM: soft lean mass, FFM: fat-free mass, SMM: skeletal muscle mass, BMI: body mass index, PBF: percent body fat, WHR: waist-to-hip ratio, VFA: visceral fat area, WC: waist circumference, HC: hip circumference.SBP: systolic blood pressure, DBP: diastolic blood pressure, UA: uric acid, TC: total cholesterol, TG: triglyceride, HDL: high-density lipoprotein cholesterol, LDL: low-density lipoprotein, FPG: fasting plasma glucose.

Table 3 Correlations between UA and other parameters at Day 62

| Variables | Day 62 | UA (μmol/L) | | Adjustment for age and sex | |
| --- | --- | --- | --- | --- | --- |
|  |  | *r/*ρ | *P* | *r/*ρ | *P* |
| BW (kg) | 92.14±15.99 | 0.397 | 0.018 | 0.284 | **0.044** |
| BFM (kg) | 35.13±9.37 | 0.178 | 0.305 | 0.061 | 0.664 |
| SLM (kg) | 53.81±9.08 | 0.485 | 0.000 | 0.415 | **0.012** |
| FFM (kg) | 57.01±9.64 | 0.481 | 0.000 | 0.340 | **0.035** |
| SMM (kg) | 31.79±5.74 | 0.491 | 0.003 | 0.316 | **0.040** |
| BMI (kg/m^2^) | 32.66±3.83 | 0.135 | 0.438 | 0.065 | 0.639 |
| PBF (%) | 37.87±5.55 | -0.095 | 0.588 | -0.017 | 0.918 |
| WHR (%) | 0.98±0.07 | 0.289 | 0.092 | -0.101 | 0.504 |
| VFA (cm^2^) | 164.44±43.84 | 0.112 | 0.521 | 0.012 | 0.993 |
| WC (cm) | 107.77±12.74 | 0.338 | 0.050 | 0.038 | 0.803 |
| HC (cm) | 109.30±6.49 | 0.340 | 0.049 | 0.148 | 0.295 |
| SBP (mmHg) | 122.22±13.86 | 0.285 | 0.114 | 0.059 | 0.685 |
| DBP (mmHg) | 74.91±11.77 | 0.238 | 0.190 | 0.047 | 0.750 |
| TC (mmol/L) | 4.77±0.84 | 0.069 | 0.697 | 0.086 | 0.496 |
| TG (mmol/L)^†^ | 1.78(1.39, 2.39) | 0.322 | 0.064 | 0.025 | 0.844 |
| HDL (mmol/L) | 1.03±0.15 | -0.165 | 0.352 | -0.066 | 0.620 |
| LDL (mmol/L) | 2.67±0.71 | 0.083 | 0.640 | 0.098 | 0.433 |
| FPG (mmol/L) | 4.58±0.62 | 0.085 | 0.633 | -0.009 | 0.945 |

Values are presented as mean ± SD for normally distributed variables and as median (Q1, Q3) for non-normally distributed variables. For normally distributed variables, pearson correlation coefficient (*r*) was used; for non-normally distributed variables (TG), spearman rank correlation coefficient (ρ) was used (marked with †). BW: body weight, BFM: body fat mass, SLM: soft lean mass, FFM: fat-free mass, SMM: skeletal muscle mass, BMI: body mass index, PBF: percent body fat, WHR: waist-to-hip ratio, VFA: visceral fat area, WC: waist circumference, HC: hip circumference.SBP: systolic blood pressure, DBP: diastolic blood pressure, UA: uric acid, TC: total cholesterol, TG: triglyceride, HDL: high-density lipoprotein cholesterol, LDL: low-density lipoprotein, FPG: fasting plasma glucose.

Table 4 Correlations between changes in UA and changes in body composition parameters across different phases

| Variables | Relative changes  (Day16-Baseline) | UA (μmol/L) | | Relative changes  (Day32-Day16) | UA (μmol/L) | | Relative changes  (Day62-Day32) | UA (μmol/L) | |
| --- | --- | --- | --- | --- | --- | --- | --- | --- | --- |
|  |  | *r* | *P* |  | *r* | *P* |  | *r* | *P* |
| BW(kg) | -3.97±1.83 | **-0.410** | **0.015** | -2.32±1.85 | 0.241 | 0.822 | -0.32±2.50 | 0.293 | 0.099 |
| BFM(kg) | -2.63±1.39 | -0.046 | 0.802 | -1.79±1.49 | 0.253 | 0.163 | -0.83±1.73 | **0.389** | **0.025** |
| SLM(kg) | -1.31±1.16 | **-0.340** | **0.025** | -0.49±1.19 | -0.224 | 0.218 | 0.46±1.19 | 0.003 | 0.987 |
| FFM(kg) | -1.34±1.24 | -0.324 | 0.062 | -0.53±1.27 | -0.217 | 0.233 | 0.51±1.25 | -0.010 | 0.955 |
| SMM(kg) | -0.76±0.73 | -0.267 | 0.139 | -0.40±0.70 | -0.122 | 0.505 | 0.23±0.71 | 0.066 | 0.716 |
| BMI(kg/m^2^) | -1.47±0.69 | -0.025 | 0.892 | -0.81±0.72 | 0.053 | 0.773 | -0.17±0.98 | 0.240 | 0.179 |
| PBF(%) | -1.04±0.97 | 0.028 | 0.877 | -1.03±1.22 | 0.341 | 0.056 | -0.79±1.18 | **0.408** | **0.018** |
| WHR(%) | -0.01±0.02 | -0.295 | 0.101 | -0.01±0.02 | 0.092 | 0.616 | -0.01±0.03 | **0.407** | **0.019** |
| VFA(cm^2^) | -11.71±6.29 | -0.164 | 0.369 | -8.28±7.87 | 0.218 | 0.231 | -4.53±8.96 | **0.492** | **0.004** |
| WC(cm) | -3.26±1.83 | **-0.376** | **0.034** | -2.40±2.09 | 0.194 | 0.287 | -1.11±3.03 | **0.499** | **0.004** |
| HC(cm) | -3.06±7.57 | -0.122 | 0.504 | -1.36±1.13 | 0.190 | 0.298 | -0.35±1.56 | 0.180 | 0.324 |

Values are reported as the mean±SD. BW: body weight, BFM: body fat mass, SLM: soft lean mass, FFM: fat-free mass, SMM: skeletal muscle mass, BMI: body mass index, PBF: percent body fat, WHR: waist-to-hip ratio, UA: uric acid, VFA: visceral fat area, WC: waist circumference, HC: hip circumference.

Figure 1. Flow chart of participants through the study.

Figure 2. Schematic overview of the study design.

Participants underwent a 62-day ADMF protocol consisting of a 32-day HCFP with stepwise caloric restriction (2/3, 1/2, 1/3, and 1/4 of baseline daily intake over four 8-day stages) and a 30-day LCFP with 500-600 kcal/day on fasting days. Anthropometry measurements and blood samples were collected at Baseline (Day 0), Day 16, Day 32, and Day 62. ADMF, alternate-day modified fasting; HCFP, high-controlled fasting phase; LCFP, low-controlled fasting phase.

Figure 3. Longitudinal changes in body weight across the 62-day intervention.

Data are presented as mean ± SD at four timepoints: baseline, Day 16 (midpoint of HCFP), Day 32 (endpoint of HCFP), and Day 62 (endpoint of LCFP). *Significant difference (*P*<0.05) compared with the changes during HCFP (paired sample *t* test). BW, body weight; SD, standard deviation; HCFP, high-controlled fasting phase; LCFP, low-controlled fasting phase.

Figure 4. Changes in weight and body composition during the two periods.

Values are reported as the mean±SD. *Significant difference (*P*<0.05) compared with the changes during HCFP (paired sample *t* test). Values are reported as the mean±SD. BW: body weight, BFM: body fat mass, SLM: soft lean mass, FFM: fat-free mass, SMM: skeletal muscle mass,WC: waist circumference, HC: hip circumference; SD, standard deviation; HCFP, high-controlled fasting phase; LCFP, low-controlled fasting phase.

Figure 5. Changes in blood pressure and metabolic indices during the two periods.

Values are reported as the mean±SD. *Significant difference (*P*<0.05) compared with the changes during HCFP (paired sample *t* test or rank-sum test). Values are reported as the mean±SD or median (1st-3rd quartile). SBP: systolic blood pressure, DBP: diastolic blood pressure, UA: uric acid, HDL: high-density lipoprotein cholesterol, FPG: fasting plasma glucose; SD, standard deviation; HCFP, high-controlled fasting phase; LCFP, low-controlled fasting phase.
